# Effect of cellular aging on memory T-cell homeostasis

**DOI:** 10.3389/fimmu.2022.947242

**Published:** 2022-08-08

**Authors:** Arpit C. Swain, José A.M. Borghans, Rob J. de Boer

**Affiliations:** ^1^ Theoretical Biology, Utrecht University, Utrecht, Netherlands; ^2^ Center for Translational Immunology, University Medical Center Utrecht, Utrecht, Netherlands

**Keywords:** homeostatic regulation, T cell, mathematical modelling, competitive exclusion, Memory attrition, cellular aging

## Abstract

The fact that T-cell numbers remain relatively stable throughout life, and that T-cell proliferation rates increase during lymphopenia, has led to the consensus that T-cell numbers are regulated in a density-dependent manner. Competition for resources among memory T cells has been proposed to underlie this ‘homeostatic’ regulation. We first review how two classic models of resource competition affect the T-cell receptor (TCR) diversity of the memory T-cell pool. First, ‘global’ competition for cytokines leads to a skewed repertoire that tends to be dominated by the very first immune response. Second, additional ‘cognate’ competition for specific antigens results in a very diverse and stable memory T-cell pool, allowing every antigen to be remembered, which we therefore define as the ‘gold-standard’. Because there is limited evidence that memory T cells of the same specificity compete more strongly with each other than with memory T cells of different specificities, i.e., for ‘cognate’ competition, we investigate whether cellular aging could account for a similar level of TCR diversity. We define cellular aging as a declining cellular fitness due to reduced proliferation. We find that the gradual erosion of previous T-cell memories due to cellular aging allows for better establishment of novel memories and for a much higher level of TCR diversity compared to global competition. A small continual source (either from stem-cell-like memory T-cells or from naive T-cells due to repeated antigen exposure) improves the diversity of the memory T-cell pool, but remarkably, only in the cellular aging model. We further show that the presence of a source keeps the inflation of chronic memory responses in check by maintaining the immune memories to non-chronic antigens. We conclude that cellular aging along with a small source provides a novel and immunologically realistic mechanism to achieve and maintain the ‘gold-standard’ level of TCR diversity in the memory T-cell pool.

## Introduction

It is well-accepted among immunologists that homeostatic mechanisms are crucial in regulating immune cell numbers. T-cell homeostasis is the phenomenon by which the T-cell population maintains its relatively stable numbers, despite considerable perturbations, such as a decline in thymic output with age and repeated exposure to antigenic challenges (1). The maintenance of CD8^+^ memory T cells generated during acute immune responses is largely cytokine-dependent, although some studies suggest that it also requires interaction with major histocompatibility complex (MHC) molecules, albeit without cognate antigen (1–7). In a lymphopenic host, increased homeostatic (density-dependent) T-cell proliferation drives the expansion of memory T cells (6, 8). Homeostasis need not be perfect, as in both mice and humans, depleted T-cell pools do not always recover to normal levels (9, 10). Notably, after autologous stem-cell transplantation, even patients with reconstituted T-cell pools experienced significantly increased T-cell proliferation and loss rates when compared to healthy age-matched controls (11). These studies highlight our incomplete understanding of the homeostatic process. A better understanding of T-cell homeostasis is central to understanding the long-term maintenance of immunological memory.

CD8^+^ memory T cells compete for the same cytokines. Interestingly, the maintenance of chronic immune responses to persisting pathogens is not only dependent on their interaction with cognate antigen (12), but also on the same cytokine(s) that the memories from acute responses depend on (5, 12–16). Due to the dependence of all memory T cells on the same resource(s), every new immune response disrupts the homeostatic balance. Responses with superior proliferative capacity (e.g., due to a higher affinity for a resource) outcompete other responses dependent on the same resource (17–20). This competition among memory T cells from different immune responses leads to moderate to severe attrition of existing memory T cells, and has been noted multiple times using both repeated vaccinations with heterologous viruses, as well as in prime-boost immunization strategies in mouse experiments (21–25). Early studies have postulated this attrition to be an effect of limited ‘space’ by showing that the total memory T-cell pool remained constant in size after consecutive infections (21, 22). Interestingly, a few recent studies report an increase in the total memory T-cell pool upon successive infections (24, 25), suggesting weaker attrition of existing memories, leading to a more diverse memory T-cell repertoire.

The mechanisms governing the maintenance of T-cell memory remain unclear. Mathematical modelling studies have assumed that memory T cells undergo global and cognate competition, concepts that correspond to well-studied ideas of inter-species and intra-species competition for resources in ecology. In mouse models, competition for cytokines, antigens, and ‘space’ in antigenic or survival niches, have been held responsible for the attrition of existing memory T cells (2, 15, 21, 22, 26–29). Competition for cytokines or physical niches leads to a non-cognate, i.e., ‘global’, form of competition between memory T cells (2, 26). Conversely, antigen-dependent competition, is confined to all cells that recognize the same cognate antigen (we refer to this as ‘cognate’ competition) (20, 27, 28, 30, 31). Low-level reactivation by cognate antigens has been suggested to have a positive effect on memory T-cell maintenance and homeostasis (22, 29, 32, 33). Several mathematical modelling studies have discussed the implications of global and cognate competition (20, 28–31, 34, 35). It was shown that global competition leads to competitive exclusion of all but the T-cell clone with the highest affinity for the resources the cells are competing for (20, 35). Global competition can be further regulated by cognate competition, even in the presence of persistent antigenic stimulation, due to competition among memory T cells sharing the same specificity (20, 28, 31, 34). Other modelling studies showed that fratricide among memory T cells crowding around the same antigen-presenting cells (APCs) can give rise to cognate competition among them, due to Fas-FasL mediated apoptosis, which provides a mechanism through which memory T-cell pools can be regulated (29, 30). Therefore, succinctly put, most current literature exploring the mechanisms underlying memory T-cell homeostasis fits in either the global or the cognate competition framework.

In this article, we propose cellular aging as an alternative mechanism that may play a role in T-cell homeostasis. That cells age is irrefutable (36–38). For instance, cellular aging has implications in cancer (37–41), and vaccination of the elderly is affected by the poor responsiveness of their aged T cells (40–47). Yet, due consideration has not been given to the aging of cells in models of memory T-cell homeostasis. Traditionally, models of T-cell homeostasis assume that cells can perform an infinite number of cell divisions, and are bounded only by the resources available at the time (29). However, a cell’s inherent division and loss rates change over time due to age, differentiation stage and division history (48–53). We, therefore, investigate the role that cellular aging may play in homeostasis and the long-term maintenance of T-cell memory.

We start with a review of the existing global and cognate competition models of T-cell homeostasis, and later move on to explore the additional effect of cellular aging on the maintenance of memory T cells. Throughout the article, we use previously described attributes of the memory T-cell pool (e.g., attrition of existing memories and expanding population size) as guides to ascertain the suitability of these three different models in generating realistic memory T-cell pools. We demonstrate that, contrary to our intuition, cellular aging helps maintain memory T-cell diversity for extended periods of time. Further, we observe that a small source, from either stem-cell-like memory T cells or from naive T cells, together with cellular aging is sufficient to maintain a diverse memory T-cell repertoire that is robust to the presence of dominant competitors (e.g., chronic immune responses to persistent pathogens). We also discuss the potential disadvantages of longevity of memory T cells, and the effect of cellular aging on the phenotypic composition of the memory T-cell pool.

## Models of memory T-cell maintenance

Three different mathematical models for the maintenance of memory T cells were defined. To keep the models simple, we considered three elements: (a) a source into the memory T-cell population (if any), (b) the most fundamental processes of any population of cells, i.e., cell division and cell death, and (c) the resources that have been demonstrated to be essential for the maintenance of memory T cells, i.e., cytokines such as IL-15, and antigens (for chronic responses). We consider both acute and chronic immune responses, where all memory T cells depend on the same homeostatic cytokine(s).

### Global competition model

**Figure 1:**
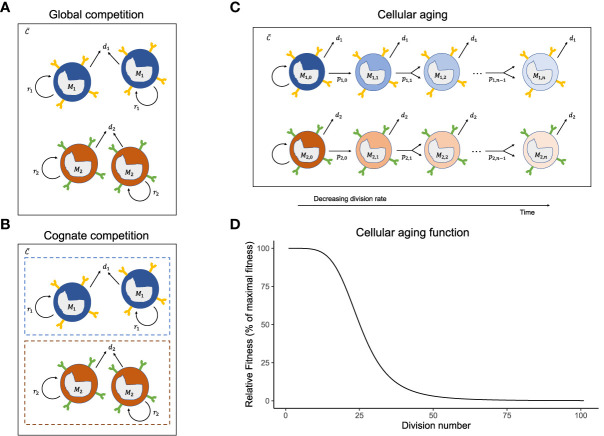
Mechanisms of homeostatic maintenance of memory T-cells. The cartoons of the **(A)** global competition, **(B)** cognate competition, and **(C)** cellular aging models showing the memory T-cell pool with two immune responses, *M*_1_ and *M*_2_ . The different immune responses can have different division rates, *p*_*i*_, and death rates, *d*_*i*_, and share the same cytokines, *C* . On top of the competition for shared global resources (cytokines) **(A)**, the cells of an immune response either compete with cells of the same specificity, leading to cognate competition **(B)** or lose their potential to divide with each cell division because of cellular aging **(C)**. The cellular aging function shows the drop in the relative fitness of a T-cell population as it divides **(D)**.

The cytokines, *C* (concentration, in mol/L), have a steady source, *σ* (in mol/L/day), from the stromal cells located across the body of a host (54, 55). Although there are other, transient sources of cytokines during inflammation, stromal cells are the major contributors during homeostatic circumstances (16, 54, 56). These cytokines are either utilized by memory T cells in a fixed amount, *ϵ* , during each cell division, or are degraded at a rate *δ* /day (Equation 1a). The dependence of all memory T cells on the same growth resource (cytokines), *C* , gives rise to global competition among the cells. The sizes of the memory T-cell populations, *M*_*i*_ , in a host having experienced *n* different antigen-specific immune responses are given by (Equation 1b, **Figure 1A**):


(1a) $$\frac{dC}{dt}=\sigma -\delta C-\epsilon \frac{C}{h+C}\sum_{i}p_{i}M_{i}$$

(1b) $$\frac{dM_{i}}{dt}=\;s_{i}+\;p_{i}M_{i}\frac{C}{h+C}-d_{i}M_{i}$$

where *i*=1,…,*n* (here *n* is the number of unique antigens encountered sequentially), and *h* is the cytokine concentration at which the homeostatic (i.e., density-dependent) proliferation rate is half-maximal estimate taken from (58). Cells of immune response *M*_*i*_ have a death rate *d*_*i*_, and a maximal homeostatic proliferation rate *p*_*i*_ (also referred to as the fitness). The source term, *s*_*i*_, defines the daily influx of memory T cells, which could be either from infrequent divisions of stem-cell-like memory T cells in the bone marrow, or from antigen-driven expansion of naive T cells (only for chronic responses) (59, 60). T cells downregulate their T-cell receptors after interactions with cytokines and are thus ‘non-greedy’ consumers of cytokines (61). Therefore, cytokine consumption was modelled to be proportional to proliferation of the memory T-cell population (Equation 1). The effective homeostatic division rate of memory T cells was set by a saturation function of the global cytokine concentration. An inverse dependence of the death rate on the cytokine concentration would give similar qualitative results (simulations not shown).

We assume that the timescales for production and degradation of cytokines are much faster than the timescales for division and death of memory T cells. Therefore, we consider the cytokines to be in quasi-steady state (Equation 2):


(2) $$C=\frac{-\left(h+\gamma \sum_{i}p_{i}M_{i}-1\right)+\sqrt{{\left(h+\gamma \sum_{i}p_{i}M_{i}-1\right)}^{2}+4h}}{2}$$

The cytokine concentration was normalized to its maximal concentration (i.e., we set $\frac{\sigma }{\delta }=1$ ), and $\gamma =\frac{\epsilon }{\delta }$ was set to 10^−6^ so that the size of the memory T-cell pool was in the order of 10^7^.

### Cognate competition model

Global competition due to sharing of growth resources leads to competition between different immune responses. Cognate competition defines the competition among the memory T cells generated during the same immune response, i.e., cells sharing the same antigen specificity (but not necessarily the same T-cell receptor). For ‘acute’ immune responses to pathogens that are eliminated, cognate competition has been proposed to follow from limited ‘space’ in specific survival niches in the bone marrow (57), or from limited availability of cross-reactive antigens (27, 28, 30). Allowing for both global and cognate competition (**Figure 1B**), the memory T-cell pools resulting from *n* different immune responses can be given by:


(3a) $$\frac{dC}{dt}=\sigma -\delta C-\epsilon \frac{C}{h+C}\sum_{i}\frac{p_{i}M_{i}}{1+\beta_{i}M_{i}}$$

(3b) $$\frac{dM_{i}}{dt}=s_{i}+\frac{p_{i}M_{i}}{1+\beta_{i}M_{i}}\frac{C}{h+C}-d_{i}M_{i}$$

for *i*= 1, 2, …, 100 antigens, and where all memory T cells specific for antigen *i* are considered to have a similar affinity for that antigen.

This extends the global competition model (Equation 1) with a cognate competition parameter, *β*_*i*_ , defining the size of the *M*_*i*_ population at which its division rate halves (which happens when *M*_*i*_=1/*β*_*i*_). All other parameters remain the same, i.e., we have in fact added an intra-specific competition term to the global competition model. As *h* was estimated before (58), the parameter *β*_*i*_ was used to tune the relative strength of the global and cognate competition. Note that global competition weakens as *h*→0 and that cognate competition declines as *β*_*i*_→0. Again, due to the very different turnover timescales of cytokines and memory T cells, the cytokines were assumed to be in quasi-steady state:


(4) $$C=\frac{-\left(h+\gamma \sum_{i}\frac{p_{i}M_{i}}{1+\beta_{i}M_{i}}-1\right)+\sqrt{{\left(h+\gamma \sum_{i}\frac{p_{i}M_{i}}{1+\beta_{i}M_{i}}-1\right)}^{2}+4h}}{2}$$

### Cellular aging model

The properties of a cell may change with cell division. It is well-known that telomere shortening during division stunts a cell’s ability to divide forever (62–64). However, in the global and cognate competition models, the cells have a constant fitness (*p*_*i*_) and, by not aging, can expand indefinitely. To account for cellular ageing, we rewrote the global competition model (Equation 1) into a division-indexed model, where we used *j*=1,…,*m*, for the number of divisions a cell has completed (i.e., *j* is the ‘generation’ number of a cell, and *m* is the maximal number of divisions it can go through, which is commonly referred to as the ‘Hayflick limit’). In our simulations, *m*=100 was chosen to be large enough so that in practice a cell never reaches its Hayflick limit. The model for the number of cells specific to antigen *i* in the *j* ^th^ division, *M*_*i*,*j*_ (see **Figure 1C**), is given by:


(5a) $$\frac{dM_{i,j}}{dt}=s_{i,j}+2p_{i,j-1}M_{i,j-1}\frac{C}{h+C}-d_{i}M_{i,j}-p_{i,j}M_{i,j}\frac{C}{h+C}$$

(5b) $$p_{i,j}=\frac{p_{i,1}}{1+{\left(j/k\right)}^{5}}$$

where *p*_*i*,*j*_ is the maximal homeostatic division rate of cells specific for antigen *i* that have completed *j* divisions; *k*=25 marks the generation number where *p*_*i*,*j*_=*p*_*i*,1_/2 ; and $M_{i}=\sum_{j}M_{i,j}$ defines the total number of cells in the *i* th immune response. For the special case where the division rates remain independent of the division number, i.e., when *p*_*i*,*j*_ = *p*_*i*_ (i.e., *k*→*∞*), this model is identical to the global competition model (it would only track the division histories of cells). Otherwise, division rates decline with the division number (Equation 5b, **Figure 1D**).

Since in the scenarios with a source, each memory T-cell population was assumed to be seeded every day with *s*_*i*_ cells, we described their division history with a Poisson distribution, i.e.,


(6) $$s_{i,j}\;=s_{i}\frac{\mu^{j}e^{-\mu }}{j!}$$

Here, *μ* is the average generation number of a precursor population formed during the expansion phase. It is defined as *μ*=2*λτ*, where *τ* is the typical length of the expansion phase (in days), and *λ*=2/day is the rate of division during the expansion phase (65). Two variations of the age-distribution of the source were modelled: ‘young’ cells (*μ*=1, or *τ*=0.25 days, i.e., cells that became quiescent after having completed one division, on average, during the expansion phase) or ‘old’ cells (*μ*=20, or *τ*=5 days, i.e., cells that divided extensively throughout the expansion phase). For simplicity, the T-cell death rate was kept the same across division numbers. Qualitatively similar results were found when implementing an increase in the death rate with increasing division number (simulations not shown). However, as less differentiated cells possess a higher expansion potential (66, 67), we chose to decrease the division rate with the division number.

### Chronic responses

To model chronic responses, we introduced extra terms specific to only chronic responses. As chronic responses are subject to additional proliferative signals due to their interaction with antigen (17), chronic immune responses have an additional maximal antigen-driven proliferation rate, *ρ*_*i*_, and a cognate (antigen-driven)-competition parameter, *g*_*i*_. The models allowing for chronic responses are, therefore, extensions of the models defined above. In case of chronic immune responses, the global and cognate competition models described by equations (1) and (3) are extended with a second proliferation term:


(7a) $$+\frac{\rho_{i}M_{i}}{1+g_{i}M_{i}}$$

The cellular aging model requires two terms to achieve a similar extension:


(7b) $$+\frac{2\rho_{i,j-1}M_{i,j-1}}{1+g_{i}M_{i}}-\frac{\rho_{i,j}M_{i,j}}{1+g_{i}M_{i}}$$

with


(7c) $$\rho_{i,j}=\frac{\rho_{i,1}}{1+{\left(j/k\right)}^{5}}$$

The cognate competition parameter, *g*_*i*_, defines the strength of the cognate competition among memory T cells specific for antigen *i*. Notice that the antigen-driven proliferation rate follows the same cellular aging function as the homeostatic (density-dependent) proliferation rate. The quasi-steady state expressions for the cytokines do not change as we assume that cell division due to stimulation by cognate antigen does not depend on the cytokine concentration.

### Parameter choices

For a fair comparison across the models, we used the same parameter values throughout this manuscript. CD8^+^ memory T cells were found to be maintained at steady state with an inter-mitotic interval of ~50 days in an adoptive transfer experiment of LMCV-specific CD8^+^ memory T cells into naive mice after being CFSE labelled (3). Reports of in-vivo deuterium labelling of non-specific CD8^+^ memory T cells supported this time scale by showing that the CD8^+^ memory T-cell pool is renewed, on an average, every ~66 days (52). Therefore, the death rate of memory T cells, *d*_*i*_, was set to 0.02/day, irrespective of the immune response. The maximal homeostatic proliferation rate, *p*_*i*_, of 0.5/day and the coefficient for global competition, *h*, of 10^−5^ were estimated based on temporal data of murine memory CD8^+^ T cells (58). As all cells in the models have the same expected life span of 50 days, and differ only in their maximum homeostatic proliferation rate, *p*_*i*_, we also refer to this proliferation rate as the ‘fitness’ of the immune response. Disparate immune responses differ in their fitness values. In the simulations, the fitness values were drawn from a normal distribution with a mean of 0.5 and a standard deviation of 0.05. The effective proliferation rate decreases as the memory T-cell pool increases and will approach *d*_*i*_=0.02 when the memory of a particular response is at steady state. The coefficient for cognate competition, *β*_*i*_ (in the cognate competition model) and *g*_*i*_ (for chronic responses), were set to be 10^−6^ and 5×10^−5^ for all *i*, respectively, so that the total mouse memory CD8^+^ T-cell pool was realistically in the order of 10^7^ cells.

### Simpson’s Diversity Index

Simpson’s diversity index has many variations. Here, we used a variation that provides an intuitive interpretation of the diversity in the memory T-cell pool. The index, based upon the relative abundances, fi=Mi∑iMi , of all immune responses, gives an indication of the effective number of immune responses in a population. The index is defined as


(8) $$\frac{1}{\sum_{i=1}^{n}f_{i}^{2}}$$

A memory T-cell pool with an index of *n* denotes a pool with *n* evenly abundant immune responses whereas an index of 1 denotes a scenario with a single, dominant immune response in the pool of immune responses.

## Results

Three models of homeostasis were formulated based on the different competition schemes: global competition (**Figure 1A**), cognate competition (**Figure 1B**) and cellular aging (**Figure 1C**, see the Models section). The global and cognate competition models are conventional models differing only in the absence or presence of intra-specific competition among memory T cells, respectively. The cellular aging model is a novel variant of the global competition model, which we propose as an alternative because there is limited evidence for cognate (intra-specific) competition among memory T cells. As this manuscript focuses on the long-term maintenance of the memory T-cell pool, we abstained from modelling the short expansion phase after an antigenic challenge. Instead, in all the simulations, we assumed that after the introduction of each antigen, the memory T-cell pool is expanded with a random number of cognate memory T cells (drawn from a normal distribution centred around 10^5^ with a 10% standard deviation), to model the beginning of a new memory phase. The simulations below reflect CD8^+^ T cells in a representative mouse (i.e., parameter values used are specific to mice).

**Figure 2:**
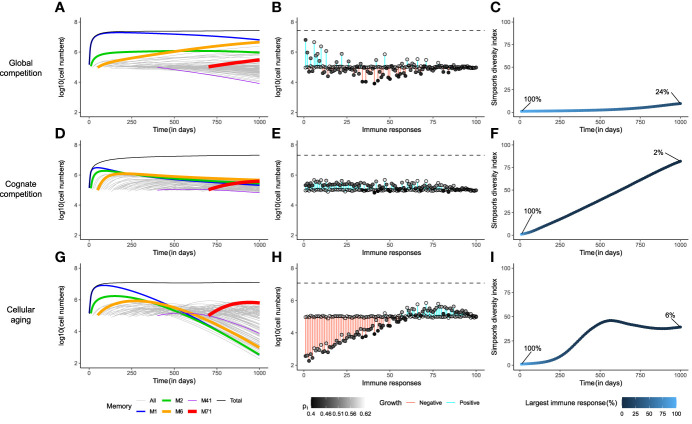
Cellular aging improves the diversity of the memory T-cell pool. Comparison of the three mechanisms for homeostatic maintenance of memory T cells showing the temporal dynamics (Panels **A**, **D**, **G**); the distribution of the sizes of all memory T-cell responses at day 1000, along with their expansion (blue) or contraction (red, indicated by the vertical bars) with respect to their initial value (indicated by the open circles) i.e., *M*_*i*_(1000)−*M*_*i*_(10×(*i*−1)) , as well as their maximal homeostatic proliferation rate (indicated by the shading of the filled circles) **(B, E**, **H)**; and the Simpson’s diversity index of the T-cell repertoire over time **(C**, **F**, **I)**. The models were simulated for 1000 days. In panels **(A, D, G)**, the thickness of the coloured lines denotes the fitness of the immune response. The dashed lines in panels **(B, E, H)** depict the size of the total memory T-cell pool on day 1000. In these simulations, the memory T-cell pool consists of acute immune responses only.

To realize the effect of the three different mechanisms on the immune dynamics over a simulated mouse’s lifetime, we recorded and compared model simulations over 1000 days (**Figure 2**). To this end, the host was successively exposed to 100 different antigens that gave rise to 100 acute immune responses with different fitness levels (see Models for details). The 100 antigens were introduced over 1000 days in 10-day time intervals from day 0 until day 990. The number of an immune response marks the time point at which (and the antigen by which) it was triggered. For example, immune response *M*_*i*_ was triggered by antigen *i* on day 10×(*i*−1) .

### Under global competition, the memory T-cell pool is dominated by a single immune response

The cytokine IL-15 is thought to be necessary for the expansion and maintenance of all memory T cells, thereby leading to global competition between the cells (20), irrespective of their antigen specificity (**Figure 1A**; see Models). In such a setting, the immune memory to the first encountered antigen, *M*_1_, in a new-born mouse, expanded to fill up the memory T-cell pool almost entirely, simply by the virtue of being the only immune response depending on the abundantly available growth factor (**Figure 2A**). The size of *M*_1_ increased, unabated, until the death in the population balanced the reduced growth of the population due to the depleted cytokine availability (i.e., $p_{1}\frac{C}{h+C}=d_{1}=0.02$ ). *M*_1_ started to decline only when the mouse was exposed to enough antigens of comparable, or higher, fitness (*M*_2_ , *M*_6_ , *M*_71_). Nevertheless, *M*_1_ dominated the memory T-cell pool almost throughout the entire lifetime of the mouse, because the rate of exclusion was very slow. At steady state, the actual division rate of the memory T cells with the highest fitness was close to their death rate *d*_*i*_=0.02 . The division rate of memory cells with 10% lower fitness (corresponding to the standard deviation of our distribution) would then be 10% lower, leading to a net loss rate of just 0.002 per day. With a half-life of about a year, it would therefore take longer than the life-span of a mouse to lose a large population of specific memory T cells, even for T cells with a relatively low fitness.

The establishment of new memories became challenging when the memory T-cell pool was nearly saturated. Immune responses with low fitness (e.g., *M*_41_ ) declined immediately upon introduction, due to the competitive pressure exerted by existing, fitter, immune responses (**Figure 2A**). Interestingly, even though existing immune responses went through attrition upon exposure to new antigens, the total memory T-cell pool showed modest growth over time (**Figure 2A** and **Supplementary Figure 1A**). This early signature of an increasing memory T-cell pool is in line with observations from previous studies on specific antigen-free laboratory mice (24, 25). In the very long run, global competition for cytokines dictated the survival of only the fittest immune response (**Supplementary Figure 1B**). Although such a scenario is disconcerting, competitive exclusion of less fit immune responses need not be achieved in a mouse’s lifetime (**Figure 2A**), as long as cells are relatively long-lived (>50 days), and fitness differences are small.

The snapshot of the total memory T-cell pool on day 1000 revealed a clear positive dependence of the size of an immune response on its maximal homeostatic proliferation rate, *p*_*i*_ (**Figure 2B**). However, *M*_1_ occupied the largest share (24%) of the memory T-cell pool even with a relatively low *p*_*i*_, as it was the first immune response. Similarly, the fittest immune response, *M*_71_, formed only a meagre 1% of the total memory T-cell pool, as it was triggered very late in the mouse’s life (on day 700). Therefore, under global competition, the size of an immune response is determined not only by its maximal homeostatic proliferation rate but also by the time at which it was generated. The Simpson’s diversity index (see Models) offers a measure of the diversity of a population by considering both the number and the disparity in the sizes of its constituents. The diversity in the memory T-cell pool barely increased over the course of the mouse’s lifetime, evolving from a repertoire with a single immune response on day 0 to one with about 10 dominant responses, and 90 small responses, on day 1000, where the largest immune response made up as much as 24% of the total memory T-cell pool (**Figure 2C**). Therefore, global competition gives rise to a skewed memory T-cell pool in which the immunity of a host weakens over time due to the loss of less fit immune responses.

### Cognate competition leads to an evenly distributed memory T-cell pool

Co-existence of multiple species is a well-known phenomenon in ecology. In a stable environment, co-existence can be achieved through intra-specific competition. Based on this idea, previous studies in immunology have suggested the presence of specific competition among the cells participating in the same immune response, because they bind similar (cross-reactive) antigens (2, 28, 29). In this section, we study a similar cognate competition model (despite a lack of experimental support), which employs competition among cells of an immune response on top of the global competition among all cells in the memory T-cell pool (**Figure 1B**; see Models).

The additional dependence on cognate resources introduced a strict limit on the size of an immune response. Despite considerable expansion, cognate competition prevented *M*_1_ from taking over the memory T-cell pool, by limiting its size (**Figure 2D** and **Supplementary Figure 1D**). In contrast to what happened in the global competition model, the cytokine was now not depleted (not shown), as inflation of *M*_1_ was avoided. So, upon exposure to new antigens, all new immune responses expanded initially, irrespective of their maximal homeostatic proliferation rate. These expansions came at the expense of the existing immune responses but contributed to the growth of the total memory T-cell pool (**Supplementary Figure 1D**). On day 1000, the memory T-cell pool was composed of many similarly-sized immune responses (**Figure 2E**), all of which (except *M*_41_) eventually reached non-zero steady state sizes that were proportional to their fitness levels (**Supplementary Figure 1D**). The immune response with the lowest fitness, *M*_41_, declined after a short bout of expansion, as its reduced homeostatic proliferation rate (due to global resource sharing) was lower than its death rate (**Supplementary Figure 1D**). The size distribution of the immune responses showed a much stronger dependence on the values of their maximal homeostatic proliferation rate (**Figure 2E**), and therefore the size of each immune response depended much less on the time at which the response was generated. In contrast to what was observed in the global competition model, the Simpson’s diversity index of the total memory T-cell pool showed an impressive increase over time to a diverse immune repertoire, in which the largest immune response consisted of only 2% of the total memory T-cell pool (**Figure 2F**).

Notably, cognate competition among cells of an immune response gives rise to a highly diverse memory T-cell repertoire. Such a repertoire is beneficial, as it offers better protection to the host over its lifetime than the very skewed repertoire that was obtained with the global competition model. Therefore, we refer to the cognate competition model as the ‘gold-standard’ in the long-term homeostatic maintenance of almost all memories.

### Immune memories are sustained for longer periods due to cellular aging

The cognate competition model gave rise to a diverse memory T-cell pool by limiting the size of the individual immune responses. Although competition in antigenic niches has been hypothesized (2, 28, 29, 68), experimental evidence for cognate competition in the memory T-cell pool is scarce. Instead, some experimental observations have shown that interactions with cognate resources (antigens) are not required for the survival of memory T cells (2, 8). Therefore, seeking for alternative mechanisms, we hypothesized that cellular aging of T cells may limit the growth of individual memory responses, and thereby generate a diverse memory T-cell repertoire. Here, we discuss the ramifications of cellular aging, in conjunction with global competition as the homeostatic mechanism, on the maintenance of the memory T-cell pool (**Figure 1C**; see Models).

The unabated expansion of the first immune response, *M*_1_, was indeed prevented by cellular aging (**Figure 2G**). Continued antigen exposures resulted in the growth of the total memory T-cell pool, while existing immune responses underwent 1) moderate erosion due to new antigen exposures (**Supplementary Figure 1E**), and **2**) major attrition due to cellular aging (**Figure 2G**). The limited consumption of cytokine by the existing immune responses allowed new immune responses to expand. However, on the long term all immune responses eventually declined (**Figure 2G**), as they were lost due to cellular aging (**Supplementary Figure 1F**). Interestingly, the maximal homeostatic proliferation rate hardly influenced the size distribution of the immune responses (**Figure 2H**). Rather, the sizes were largely determined by the time at which the responses were generated. The most recent responses made up the majority of the memory T-cell pool, as the older a response was, the more it was eroded. The diversity within the memory T-cell pool was much larger than in the global competition model but was only half of that achieved in the cognate competition model (**Figure 2I**). The largest immune response on day 1000 occupied a mere 6% of the memory T-cell pool, compared to the inflated 24% in the global competition model. Therefore, although the cellular aging model improves upon the global competition model, it cannot generate a memory T-cell pool as diverse as the ‘gold-standard’ memory T-cell pool that results from cognate competition.

### A small source into an aging population helps to maintain a diverse memory T-cell pool

Multiple studies in the recent past have described subsets of memory T cells that have superior potential to generate other memory T-cell subsets (69). This self-sustaining population is sometimes referred to as the stem-cell-like memory T-cell population. Stem-cell-like memory T cells, generated during an acute response and residing in stromal niches in the bone marrow, could act as a slow but steady source into the circulating memory T-cell populations. We have seen that cellular aging can limit the expansion of the early memory populations, thereby reducing competition, but that the memory T-cell pool suffers in the long run, due to the eventual loss of all memories by cellular aging. The presence of a lowly-divided stem-cell-like source for each immune response would circumvent this issue. Importantly, this memory-maintaining source could also originate from circulating memory T cells due to repeated infections, from cross-reactions with other antigens, or from activation of new naive T cells (in case of persistent antigens).

**Figure 3:**
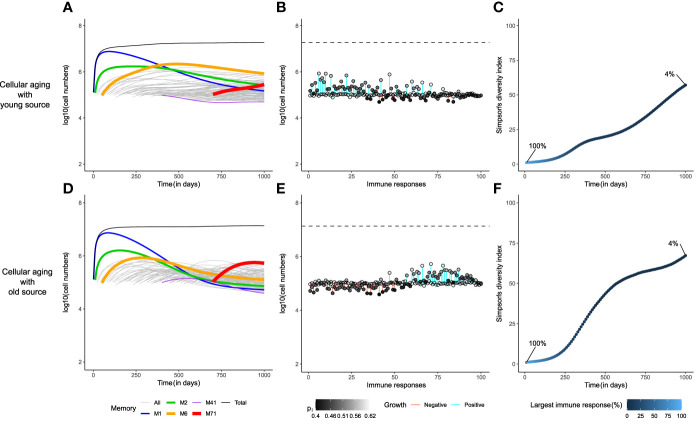
A small source helps to achieve the gold-standard diversity within the memory T-cell pool. The dynamics of the cellular aging model along with a source from either a young (upper row) or an old population (lower row). The memory T-cell pool was generated by acute immune responses only. Legends as in **Figure 2**.

Indeed, if each immune response had a small source (*s*_*i*_=100 cells/day), none of the immune responses would be lost in the long-term (**Figure 3**). As the division history of the source might affect the memory T-cell repertoire, we evaluated two different scenarios: one, where the source population turned quiescent early in the expansion phase (‘young’ source, having completed 1 division on average), and the second, where the source population had divided as much as the circulating cells before becoming quiescent (‘old’ source, having completed an average of 20 divisions). A young source sustained the growth of early immune responses for longer periods of time compared to an older source (**Figures 3A, D**). Remarkably, the size distribution of the memory T-cell pool was completely different in both cases (**Figures 3B, E**). The presence of a young source resulted in a pool dominated (in size) by earlier immune responses, whereas a source from an older population favoured the prevalence of recently generated immune responses. This effect was due to a difference in the ‘effective source’. Although both scenarios have the same source of 100 cells/day, the source from a younger population contributes more daughter cells because of their higher homeostatic proliferation rate. When the source population is old, it expands less and makes for a smaller effective source to the existing memories. Hence, with an old source the later immune responses expand more, due to weaker competition from existing immune responses, compared to the scenario with a young source. Even with such a stark difference in the immune profiles, the diversity within the memory T-cell pool was comparable in both scenarios, as none of the immune responses were lost (**Figures 3C, F**). The Simpson’s diversity index revealed that the diversity achieved by adding a stem-cell-like source to the cellular aging model was comparable to the ‘gold-standard’ diversity achieved with the cognate competition model. Notably, the presence of a source in the global competition model failed to improve its diversity, demonstrating that adding a small source is not a trivial solution to maintaining the diversity of a population (figure not shown due to its similarity to **Figures 2A–C**). Therefore, we propose that a source into an aging population is a viable alternative to the cognate competition model.

**Figure 4:**
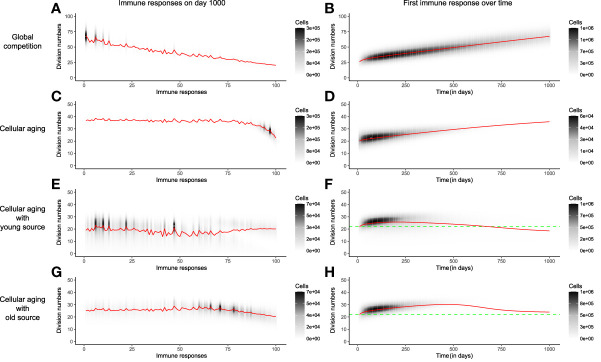
The distribution of division numbers in the memory T-cell pool is determined by the age of the source population. Comparison of the distribution of division numbers of all immune responses on day 1000 (Panels **A**, **C**, **E**, **G**), and the division history of the first immune response over time (Panels **B**, **D**, **F**, **H**), for different mechanisms. The red line plots the average generation number of an immune response. The grey and black shades show the number of cells at different division numbers. The green dotted line marks the starting division number of the first immune response. In these simulations the memory T-cell pool consists of acute immune responses only.

The distribution of the division number in the memory T-cell pool of the global competition model in the absence of cellular aging, i.e., when *p*_*i*,*j*_=*p*_*i*_, showed a large disparity in the average generation number of the immune responses, with early responses having divided 70 times on average, compared to an average of 25 divisions for recent responses (**Figure 4A**). The very first response (like all other responses) had gone through 25 divisions when seeded in the beginning of the memory phase, and subsequently accumulated 50 more divisions throughout the mouse’s life (**Figure 4B**). Even though the cells of the first immune response accrued more divisions with time, the size of the first response declined over time due to global competition from successive immune responses (**Figures 4B**, **2A**). The disparity found in the division distribution of the memory T-cell pool dropped considerably in the presence of cellular aging (**Figure 4C**). Cellular aging promoted the dominance of recent immune responses, even though they had divided less (**Figures 4C**, **2H**). The number of divisions accrued by an immune response over a mouse’s lifetime was also significantly lower due to cellular aging (**Figure 4D**). A continuous source reduced the disparity in the division distribution within the memory T-cell pool even more (**Figures 4E**, **4G**). The average generation number of the first immune response on day 1000 was lower compared to that on day 0 when the source was from a younger population but was higher than that on day 0 in case of an older source (**Figures 4F**, **4H**). Therefore, a small source counteracts the exhaustion of a population by cellular aging and the division history of the source determines the distribution of the division history within the memory T-cell pool.

### A source into an aging T-cell population maintains immune memories in the long-term even in the presence of chronic responses

Our discussion until now has focused on a memory T-cell pool containing 100 memories to pathogens that were eliminated during the ‘acute’ immune response. However, chronic immune responses to pathogens (or antigens) that persist, may pose a big challenge in maintaining the diversity of the memory T-cell pool, due to their sometimes inflationary properties (12). We assessed whether our novel model, with a memory T-cell pool going through cellular aging in the presence of a source, could maintain T-cell diversity under the competitive pressure from chronic T-cell responses. We considered a host that generated 95 acute responses and 5 chronic responses, even though only 1% of all infections are estimated to lead to chronic responses (12). Chronic responses are maintained in part by homeostatic proliferation due to IL-15, and partly due to repeated stimulation by persistent antigen (see Models).

**Figure 5:**
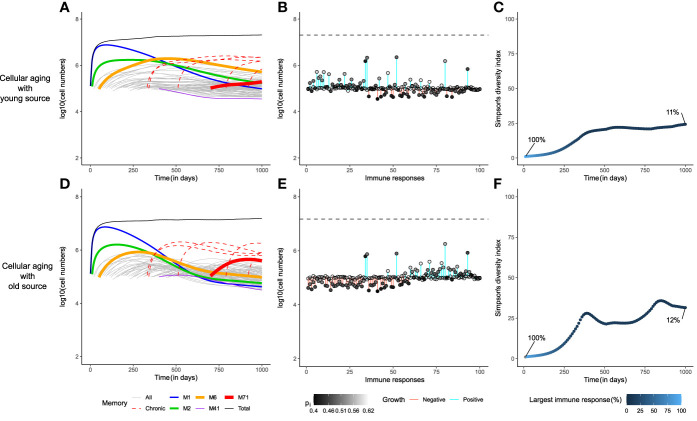
Chronic immune responses lower the long-term diversity of the memory T-cell pool. The dynamics of the cellular aging model along with a source from either a young source (upper row) or an old source (lower row). The memory T-cell pool consists of both acute and chronic immune responses shown as dashed red line. Legends as in **Figure 2**.

Chronic responses expanded to occupy a higher proportion of the memory T-cell pool than acute responses (**Figure 5**). The immune dynamics of the 95 acute responses were not severely affected by the addition of 5 chronic responses (compare **Figures 3A, D** with **Figures 5A, D**). Exposure to chronic antigens expanded the total memory T-cell pool, and the heightened competition reduced the sizes of the acute responses somewhat. The division histories and the prevalence patterns of the immune responses hardly changed (compare **Figures 3B, E** with **Figures 5B, E**, and **Supplementary Figure 2**). Like before, the division history showed that the memory T-cell pool became younger with time in the presence of a young source, which resulted in the prevalence of early immune responses. However, the diversity of the memory T-cell pool declined markedly in the presence of chronic responses as the largest immune response now made up only about 10% of the total memory T-cell pool (**Figures 5C, F**). The cellular aging model along with a source, thus, provides a robust mechanism for the long-term maintenance of memory T-cells, even though the diversity in the memory T-cell pool suffers from the presence of chronic responses.

### Long-lived memory T cells lower the diversity of the memory T-cell pool

Circulating memory T cells have been shown to be relatively short-lived (70), while memory in itself is long-lived (71, 72). In an attempt to understand why memory T cells are relatively short-lived, we studied how the lifespan of memory T cells influences the diversity of memory T-cell repertoires. In our models, the attrition of existing memories was due to the relatively short lifespans of memory T cells. We examined whether memory T cells with longer lifespans would allow for higher diversity in the memory T-cell repertoires. To precisely underline the influence of memory T-cell lifespan (without the effect of a source) on the diversity of the memory T-cell repertoire, we only considered the three basic mechanisms: global competition, cognate competition, and cellular aging, without any source. The model characteristics were compared considering a scenario where memory T cells lived 10 times longer than their estimated lifespans, i.e., *d*_*i*_=0.002/day (**Supplementary Figure 3**). The temporal dynamics corroborated the previous conclusions: *M*_1_ filled up the memory T-cell pool under the influence of global competition, while this was strongly and moderately constrained in the cases of cognate competition and cellular aging, respectively (**Supplementary Figure 3A, D, G**). Notably, none of the cases showed the loss of any immune response (**Supplementary Figure 3B, E, H**). The degree of inflation of an immune response clearly correlated with how early the response was generated. More importantly, the levels of TCR diversity in the memory T-cell pool were a lot lower when compared to the corresponding cases with short-lived memory T cells (**Supplementary Figure 3C, F, I**). When memory T cells were long-lived, the diversity in the global competition and cellular aging models barely improved over time, whereas the index for the cognate competition model was half of that with short-lived memory T cells.

Although maintenance of all encountered immune responses may be advantageous, the inflation of early memories might pose significant challenges for the efficient protection of a host. The recall response to a recent antigen is expected to be delayed when the probability of finding cognate memory T cells decreases due to the presence of inflated early responses in the pool. A considerable delay in the recall response to a large infection may even be detrimental (25). Therefore, surprisingly, storing immunological memory in short-lived memory T cells may be more beneficial, as short-lived memory T cells allow for higher diversity in the memory T-cell pool.

## Discussion

Here, we showed that cellular aging in the presence of a source population is a mechanism by which long-term maintenance of a diverse memory T-cell pool can be achieved. It preserves acute as well as chronic immune responses in the long-term and generates a diverse memory T-cell repertoire comparable to the gold-standard level of diversity generated with cognate competition. Whereas the occurrence of cognate competition is poorly supported by experimental evidence, the presence of both cellular aging and a source (from stem-cell-like memory T cells, re-activated memory T cells, cross-reactive memory T cells or newly activated naive T cells) are widely accepted. Therefore, we propose that a source into an aging population is an immunologically viable alternative to the cognate competition model.

The global competition model is prone to competitive exclusion of all but the fittest immune response. Using division and death rates of murine memory CD8^+^ T cells, we showed that although competitive exclusion may not be seen in the lifetime of a mouse, global competition would lead to unrealistically skewed memory repertoires. Moreover, the slow exclusion of memory T-cell responses in our simulations was due to the small differences in the fitness values of the different immune responses. If the fitness values of the immune responses were to differ more than the 10% standard deviation considered in our simulations, the size disparity in the memory T-cell pool would be even higher.

The cellular aging model improves upon the global competition model by limiting the size of each self-renewing memory population, which reduces the competition among them. A source into an aging memory T-cell pool sustains the diversity of the memory T-cell repertoire in the long-term. A young source favours the frequency of early memory responses, while an old source causes recent responses to be more prevalent. The memory T-cell pool eventually turns younger due to the presence of a young source. This could present a potential explanation for the observation that reconstituted T-cell pools have higher proliferation rates after autologous stem-cell transplantation (11) as the highly-divided circulating memory T cells were replaced with lowly-divided memory T cells. Moreover, if the acquisition of different memory T-cell phenotypes were correlated with the division history of a cell (73), cellular aging would have exciting implications. For example, following the linear differentiation pathway (74), the absence of a source would predict the accumulation of effector memory T cells over time, whereas the presence of a source would suggest the accumulation of central memory T cells after multiple infections. Previous studies addressing repeated vaccinations (75) have shown diverging results. Some studies showed the enrichment of memory T cells with an effector memory phenotype (33, 76), whereas other studies showed the accumulation of central memory T cells after multiple rounds of heterologous, viral vaccinations (25). Similar effects of sustained, chronic responses on the phenotypes of both bystander and specific memory T cells have also been discussed (77, 78). Further, the declining fitness of immune responses in older hosts can explain their impaired response to vaccinations (79). In view of such observations, it is extremely interesting to study the mechanisms underlying the phenotypic distribution in memory T-cell pools, and thereby the erosion of protective immunity with age.

In absence of a source, an aging population will eventually be lost. The timescale of this extinction is much longer than the lifetime of a mouse. Interestingly, erosion of early memories could be beneficial for a host to maintain memory and mount responses against recently encountered pathogens, as the relative proportion of recently generated memory T cells would increase. In a changing natural environment, the probability of getting re-exposed to a pathogen is probably higher for recently encountered pathogens than for those encountered early in life. Therefore, maintenance of recent memories could be beneficial. Along similar lines, we also showed that long-lived memory T cells, or lack of regulation (due to competition or aging) early in life, would lead to a loss of diversity in the memory T-cell pool due to inflation of early memories. Virtual memory T cells are memory-phenotype cells that originate from naive T cells due to homeostatic proliferation in the absence of cognate antigen (80, 81). If virtual memory T cells would be highly inflated due to lack of competition early in life, and/or due to long lifespans, they would severely impair the efficacy of the memory T-cell repertoire against natural infections.

Many laboratory protocols, just like our simulations, use fixed time intervals between antigen introductions, a scheme that is, of course, rather artificial. In reality, a mouse’s exposure to antigens is truly a random event. Statistically, random events follow a Poisson distribution, where the time between two consecutive events is exponentially distributed. Therefore, a mouse in its natural environment will be exposed to most unique antigens early on in its life and to relatively fewer novel antigens in its twilight years. For our models, simulations of this real world scenario led to considerably different results from the simulations presented here (**Supplementary Figure 4**). When emulating the real world scenario, the first immune response, *M*_1_ , did not take over the memory T-cell pool in any of the models, due to competition with multiple other immune responses generated early in life. The snapshots of the memory T-cell pool on day 1000 showed a more marked attrition of immune responses, under the influence of global competition and cellular aging but not under cognate competition (**Supplementary Figure 4**). If anything, our simulations showed that random antigenic exposure accelerates the competitive dynamics and reduces the diversity of the memory T-cell repertoire.

Although our simulations were primarily based on murine parameters, the concerns and results discussed here are also applicable to humans. The expected lifespan of human circulating memory CD8^+^ T cells is close to 200 days (50), which is 4 times longer than that in mice. However, the lifespan of a human is 30 times longer than that of a mouse. Thus, based upon our global competition model, one would expect early immune responses to be competitively excluded during a human’s lifetime. Both cognate competition and cellular aging with a source would alleviate this problem, the latter providing a more immunologically sound mechanism. Unfortunately, we are not aware of any literature on the effects of cellular aging of memory T cells that could be used to test the prediction of our novel model. Single-cell sequencing studies have described the change in the memory T-cell repertoire with age (82). However, these studies have focused on the age of the host rather than the age of the cell, making a comparison of these results and our predictions speculative. Recent studies using the Cre-recombinase technology (48, 53) do provide an avenue that could be exploited to delineate the age of the cell from the age of its host. Such dedicated experiments would be required to test the predictions of the cellular aging model.

Barring a few studies (83), IL-7 is often implicated in the homeostatic maintenance of memory as well as naive T cells (4, 57, 61). The competition between naive and memory T-cell populations through IL-7 has not been taken into account in this study. As the production of new naive T cells declines with age (84, 85), reduced competition for IL-7 could alleviate some of the ‘global’ competitive pressure on the memory T-cell pool, leading to a larger memory pool size, but not to an alteration in the distribution of clone sizes (i.e., not to a different memory T-cell repertoire). Similarly, a temporal change in the source of homeostatic cytokines (IL-15, IL-7, etc.) e.g., due to aging, would affect the global competition among memory T cells and change the pool size but not the repertoire. IL-15 is produced by multiple tissues (e.g., bone marrow, heart, lung, kidney, thymic epithelium) and cell types (e.g., monocytes/macrophages, blood-derived dendritic cells) (86) that are subject to alterations throughout a host’s life. Since the lifespan of circulating memory T cells has a time scale of months in mice, the division rate is expected to average over such spatial heterogeneities. In our model, the source is considered to be either from naive T cells due to new or recurrent challenges, or from stem-cell-like memory T cells. As the exposure to antigens is random, the source from naive T cells could be stochastic. Further, newly generated stem-cell-like memory T cells in the bone marrow could either increase the source to an immune response (in case of a recurrent infection) (87, 88), or decrease the source (due to competition among memory T-cells for the limited number of stromal niches) (89, 90). Thus, the source need not be constant. It would be interesting to study such model variations in future studies and to quantify their effect on the long-term maintenance of the memory T-cell pool.

The cellular aging model, like existing models for the maintenance of memory T cells, presents a simplified view of the memory T-cell pool and its maintenance. However, unlike the current gold-standard based on cognate competition, cellular aging is well-supported. Therefore, this manuscript puts forth a realistic mechanism that might underlie the observed long-lived large diversity of the CD8^+^ memory T-cell repertoire despite the relatively short lifespan of CD8^+^ memory T cells.

## Data availability statement

The original contributions presented in the study are included in the article/**Supplementary Material**. Further inquiries can be directed to the corresponding author.

## Author contributions

AS performed mathematical modelling. AS, JB, RdB designed the study. AS, JB, RdB analyzed the data and wrote the manuscript. All authors contributed to the article and approved the submitted version.

## Funding

This work was supported by an NWO grant (ALWOP.265) to RdB and an NWO Vici grant (R5732) to JB.

## Conflict of interest

The authors declare that the research was conducted in the absence of any commercial or financial relationships that could be construed as a potential conflict of interest.

## Publisher’s note

All claims expressed in this article are solely those of the authors and do not necessarily represent those of their affiliated organizations, or those of the publisher, the editors and the reviewers. Any product that may be evaluated in this article, or claim that may be made by its manufacturer, is not guaranteed or endorsed by the publisher.

## Supplementary material

Supplementary Figure 1 Global competition leads to the loss of all but the fittest immune response. Comparison of the three mechanisms (global competition, cognate competition, and cellular aging) for homeostatic maintenance of memory T cells focusing on the short-term (first 100 days in Panels **A**, **C**, **E**) and long-term (10,000 days in Panels **B**, **D**, **F**) temporal dynamics of a murine memory T-cell pool. The memory T-cell pool consists of acute immune responses only.

Supplementary Figure 2 Adding chronic responses hardly changed the division distributions. Comparison of the division distributions of all memory T-cell populations on day 1,000, and the division history of the first memory T-cell population over time. The red line plots the average generation number of an immune response. The grey and black shades show the number of cells at different division numbers. The green dotted line marks the starting division number of the first immune response. The memory T-cell pool consists of both acute and chronic immune responses.

Supplementary Figure 3 Memory T-cell pools with long-lived memory T cells are barely diverse. Comparison of three mechanisms (global competition, cognate competition, and cellular aging, without any source) for homeostatic maintenance of long-lived memory T cells. The expected lifespan of the memory T cells was set to 500 days (which is 10 times longer than their estimated lifespans (3)). The memory T-cell pool consists of acute immune responses only.

Supplementary Figure 4 Random antigen exposure aggravates the loss of immune memories due to intense competition. Comparison of three mechanisms (global competition, cognate competition, and cellular aging, without any source) for homeostatic maintenance of memory T cells when most novel pathogens appear early in life. The delay between antigen exposures was exponentially distributed, mimicking the random exposure to antigens in real life. The memory T-cell pool consists of acute immune responses only.
