# Supplementary figures and images for: Effect of cellular aging on memory T-cell homeostasis
Source: Front Immunol. 2022 Aug 8;13:947242. doi: 10.3389/fimmu.2022.947242 (PMC9429809; doi:10.3389/fimmu.2022.947242)

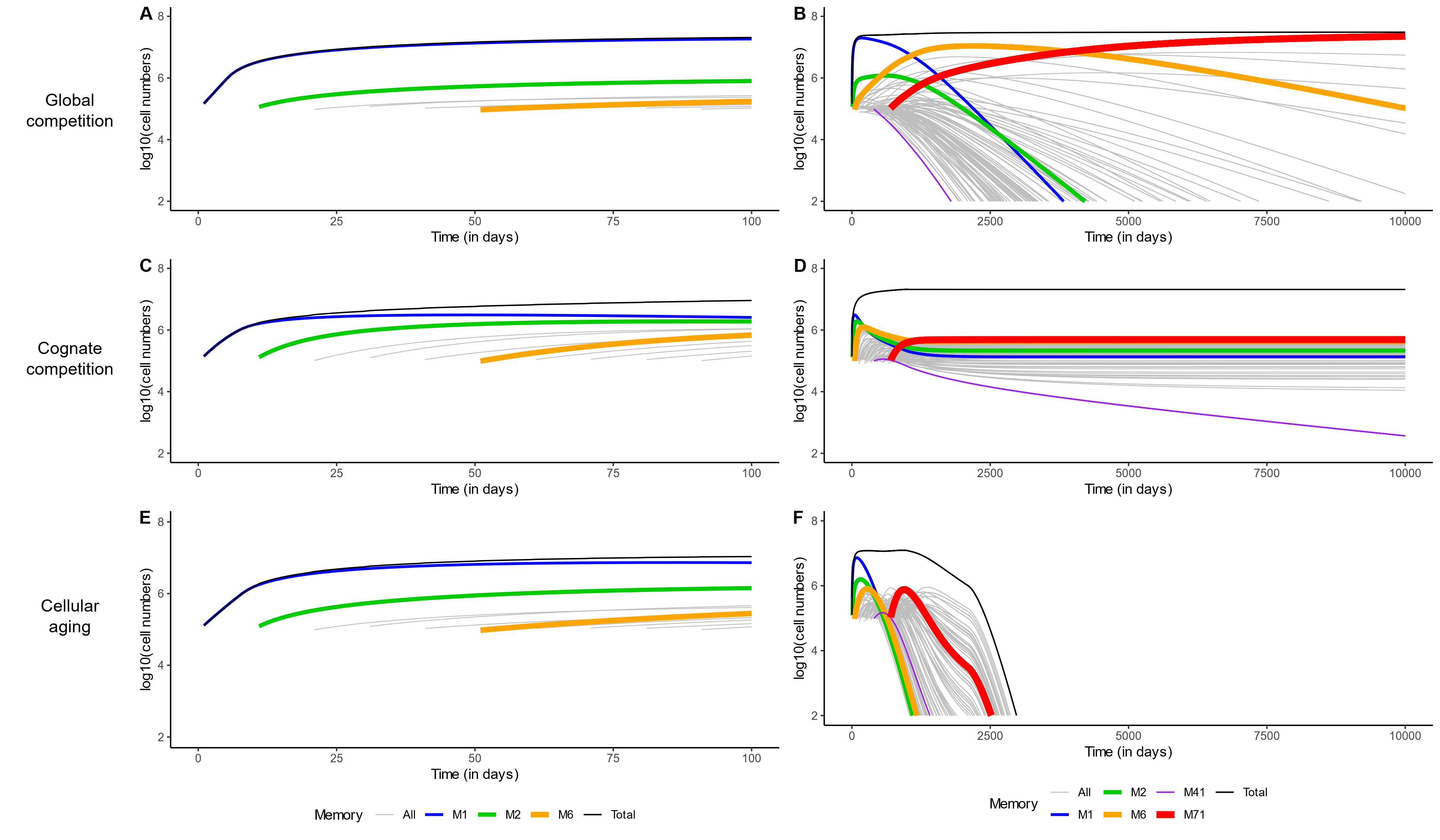

Supplement: Supplementary Figure 1 — Global competition leads to the loss of all but the fittest immune response. Comparison of the three mechanisms (global competition, cognate competition, and cellular aging) for homeostatic maintenance of memory T cells focusing on the short-term (first 100 days in Panels A, C, E) and long-term (10,000 days in Panels B, D, F) temporal dynamics of a murine memory T-cell pool. The memory T-cell pool consists of acute immune responses only. [file Image_1.tif]
